# Group 1 and group 2 hemagglutinin stalk antibody response according to age

**DOI:** 10.3389/fimmu.2023.1194073

**Published:** 2023-05-29

**Authors:** Laura Sánchez-de Prada, Iván Sanz-Muñoz, Weina Sun, Peter Palese, Raúl Ortiz de Lejarazu, José María Eiros, Adolfo García-Sastre, Teresa Aydillo

**Affiliations:** ^1^ National Influenza Center of Valladolid, Valladolid, Spain; ^2^ Department of Microbiology and Immunology, Hospital Clínico Universitario de Valladolid, Valladolid, Spain; ^3^ Department of Microbiology, Icahn School of Medicine at Mount Sinai, New York, NY, United States; ^4^ Centro de Investigación Biomédica en Red de Enfermedades Infecciosas, Instituto de Salud Carlos III, Madrid, Spain; ^5^ Global Health and Emerging Pathogens Institute, Icahn School of Medicine at Mount Sinai, New York, NY, United States; ^6^ Department of Pathology, Molecular and Cell-Based Medicine, Icahn School of Medicine at Mount Sinai, New York, NY, United States; ^7^ Department of Medicine, Division of Infectious Diseases, Icahn School of Medicine at Mount Sinai, New York, NY, United States; ^8^ The Tisch Cancer Institute, Icahn School of Medicine at Mount Sinai, New York, NY, United States

**Keywords:** influenza, stalk antibodies, influenza vaccines, age, elderly

## Abstract

**Objective:**

Antibodies elicited by seasonal influenza vaccines mainly target the head of hemagglutinin (HA). However, antibodies against the stalk domain are cross-reactive and have been proven to play a role in reducing influenza disease severity. We investigated the induction of HA stalk-specific antibodies after seasonal influenza vaccination, considering the age of the cohorts.

**Methods:**

A total of 166 individuals were recruited during the 2018 influenza vaccine campaign (IVC) and divided into groups: <50 (n = 14), 50–64 (n = 34), 65–79 (n = 61), and ≥80 (n = 57) years old. Stalk-specific antibodies were quantified by ELISA at day 0 and day 28 using recombinant viruses (cH6/1 and cH14/3) containing an HA head domain (H6 or H14) from wild bird origin with a stalk domain from human H1 or H3, respectively. The geometric mean titer (GMT) and the fold rise (GMFR) were calculated, and differences were assessed using ANOVA adjusted by the false discovery rate (FDR) and the Wilcoxon tests (p <0.05).

**Results:**

All age groups elicited some level of increase in anti-stalk antibodies after receiving the influenza vaccine, except for the ≥80-year-old cohort. Additionally, <65-year-old vaccinees had higher group 1 antibody titers versus group 2 before and after vaccination. Similarly, vaccinees within the <50-year-old group showed a higher increase in anti-stalk antibody titers when compared to older individuals (≥80 years old), especially for group 1 anti-stalk antibodies.

**Conclusion:**

Seasonal influenza vaccines can the induction of cross-reactive anti-stalk antibodies against group 1 and group 2 HAs. However, low responses were observed in older groups, highlighting the impact of immunosenescence in adequate humoral immune responses.

## 1 Introduction

The influenza virus, with three to five million severe cases and between 290,000 and 650,000 annual respiratory deaths (1), represents a major socioeconomic burden (2). Currently, the best approach to preventing infection and reducing disease severity is annual vaccination. However, influenza vaccine effectiveness is moderate, varying from 20% to 70% depending on the season. Additionally, influenza vaccines provide short-lasting and strain-specific protection (3). Most neutralizing antibodies induced by vaccination target hemagglutinin (HA), particularly the immunodominant head domain, which constantly undergoes antigenic drift by accumulating amino acid substitutions and additional glycosylation sites (4). Additionally, during some seasons, the strains contained in the vaccine do not match the circulating strain(s) due to viral evolution. Such low effectiveness makes it necessary for vaccines to be reformulated and re-administered annually (5).

The HA is the most abundant surface glycoprotein and has two major domains: the globular head (HA1) and the stalk region (HA2). The HA stalk domain is highly conserved between influenza virus strains due to functional restraints and low immune pressure (6). There a re currently 18 hemagglutinin subtypes for influenza A virus, which are classified into two phylogenetic groups based on their antigenic properties: group 1 consists of H1, H2, H5, H6, H8, H9, H11, H12, H13, H16, H17, and H18; while group 2 contains H3, H4, H7, H10, H14, and H15 (7). Antibodies against the stalk are more cross-reactive and can bind different strains of the same phylogenetic group, providing broad protection. Mechanisms of these antibodies may include impairment of viral and endosomal membrane fusion, inhibition of viral release, and interruption of HA maturation. In addition, these antibodies are functionally involved in antibody-dependent cell cytotoxicity and phagocytosis (ADCC and ADCP) and complement-dependent cytotoxicity (CDC) (8). Novel influenza vaccine designs are focused on the development of influenza vaccines that would increase the breadth and duration of protection. Some of the most advanced vaccine candidates target conserved epitopes of the HA protein, such as the subdominant stalk domain, with the aim of providing long-lasting protection against different strains and subtypes of the virus (8, 9).

The aim of our study is to investigate the level of pre-existing anti-stalk antibodies against phylogenetic groups 1 and 2, and after seasonal influenza vaccination according to age. Immunodominance profiles and antibody titers against different antigenic sites in the HA head of A(H1) that matched influenza vaccine strains were previously studied in this cohort. Classically, five antigenic sites in the head of the HA have been defined as Sb, Sa, Cb, Ca1, and Ca2 and are the main targets of the humoral response upon vaccination or infection. The first two are placed at the distal tip of each monomer, while Cb, Ca1, and Ca2 are placed proximally, near the stalk domain. The receptor binding site (RBS), where the attachment to sialic acids occurs, is located between Sb, Ca2, and Sa (10, 11). We found that the immune response was mainly directed at Sb, followed by Ca2, and that adjuvants can broaden responses to subdominant antigenic sites (12). Here we expand on our previous study and now investigate the antibody response to the stalk domain according to age.

## 2 Materials and methods

### 2.1 Patient recruitment

A total of 166 individuals were recruited from vaccination programs during the Influenza Vaccine Campaign (IVC) 2018 conducted by the Influenza Sentinel Surveillance Network of Castile and Leon (Spain) (ISSNCyL). All serum samples obtained were shipped to Mount Sinai Hospital in New York (USA) and were used to determine HA stalk-specific antibodies. Serum samples were obtained before and 28 days after vaccination and stored at −20°C in the National Influenza Centre of Valladolid (Spain) before being sent. Two seasonal influenza vaccines were used following the recommendations of the World Health Organization (WHO) for the northern hemisphere: A/Michigan/45/2015 (H1N1)pdm09-like virus, A/Singapore/INFIMH-16-0019/2016 (H3N2)-like virus, and B/Colorado/06/2017-like virus (B/Victoria/2/87 lineage) for the trivalent vaccine, and also B/Phuket/3073/2013-like virus (B/Yamagata/16/88 lineage) for the quadrivalent one. Following the recommendations for vaccination in Spain, subjects ≥65 years old received an adjuvanted trivalent influenza vaccine (ATIV) and subjects <65 years old received a quadrivalent influenza vaccine (QIV). Two patients from each group received the other group’s vaccine due to a lack of vaccine availability. Written informed consent was obtained from the participants. This research was performed according to the Declaration of Helsinki and was approved by the Ethics Committee of the East-Valladolid Health Area under the code PI 21-2314.

### 2.2 Stalk-specific antibodies

To quantify the levels of the stalk-specific antibodies, two reassortant viruses were used: a cH6/1N5 and a cH14/3N5. The first one had an HA stalk derived from the pandemic H1N1 virus (A/California/04/09) containing an exotic H6 head domain (H6N1 virus A/mallard/Sweden/81/02) and an exotic N5 (H12N5 virus A/mallard/Sweden/86/03). HA head domains were of wild bird origin, and hence no specific antibodies should be present in the patients’ serum samples. The methods and description of the generation of this virus in cell culture by using reverse genetics have been previously published (13–15). The second virus had an HA stalk derived from an H3N2 virus A/Hong Kong/4801/2014 combined with an exotic H14 head domain A/mallard/Gurjev/263/1982 and an exotic N5 from the H12N5 virusA/mallard/Sweden/86/03 (for virus generation, see the **Supplementary Appendix**). Reassortant viruses were cultured in 10-day-old embryonic chicken eggs and titered to confirm the growth and ensure they had similar hemagglutination units. Then, a purification by ultracentrifugation in a sucrose gradient was performed (**Supplementary Appendix**). Antibodies in human serum were measured using an enzyme-linked immunosorbent assay (ELISA) as described before (16) (for the ELISA protocol, see the **Supplementary Appendix**). The optical density (OD) for each well was calculated by subtracting the average background plus three standard deviations. The area under the curve (AUC) was computed using GraphPad Prism v.10 software.

### 2.3 Statistical analysis

All ELISA values were log10-transformed to improve linearity. The GMT and 95% confidence intervals (CI 95%) were computed by taking the exponent (log10) of the mean and the lower and upper limits of the 95% CI of the log10‐transformed titers. Fold rise was calculated as the ratio between days 0 and 28. GMFR was computed by taking the exponent (log10) of the mean fold rise and the lower and upper limits of the CI 95% of the log10‐transformed titers. Statistical significance was established at p <0.05. All reported p values are based on two‐tailed tests. For antibody levels, the Brown–Forsythe and Welch ANOVA test was adjusted by controlling the false discovery rate (FDR) with the two-stage linear procedure of Benjamini, Krieger, and Yekutieli for multiple comparisons, and the Wilcoxon matched pairs signed rank test was used when appropriate. All tests were performed using IBM SPSS Statistics (version 26) and GraphPad Prism (version 10).

## 3 Results

### 3.1 Human cohorts

A total of 166 individuals were recruited during the Influenza Vaccine Campaign (IVC) 2018. Two different inactivated influenza vaccines were applied according to age following Spanish recommendations: a quadrivalent influenza vaccine (QIV) in 46 subjects of 28–64 years and two subjects of 73 and 74 years old (28.9%), and an MF-59 adjuvanted trivalent influenza vaccine (ATIV) in 116 subjects ≥65 years old and two subjects of 57 years old (71.1%). To assess the presence of HA stalk-specific antibodies, vaccinees were divided according to age into four groups: <50, 50–64, 65–79, and ≥ 80 years old. Epidemiological and clinical characteristics are described in **Table 1**.

**Table 1:** Cohort description and epidemiological and clinical characteristics.

	<50 years old	50–64 years old	65–79 years old	≥80 years old
**No.**	14	34	61	57
**Age (Median, IQR)**	37.5 (29.75–45.5)	59.5 (55.0–62.25)	70.0 (68.0–74.0)	85.0 (83.0–90.0)
**Men (%)**	14.3	55.9	60.7	49.1
**Type of vaccine**	Vaxigrip	Vaxigrip	Chiromas	Chiromas
**Comorbidities (n, %)**	1 (7.1)	4 (11.8)	10 (16.4)	4 (7.0)
Diabetes mellitus	0	1	3	1
Heart disease	0	1	4	2
COPD	0	0	0	1
Immunocompromised	1	2	3	0

No, number; IQR, interquartile range; COPD, chronic obstructive pulmonary disease.

### 3.2 Anti-stalk antibodies according to age

**Figure 1:**
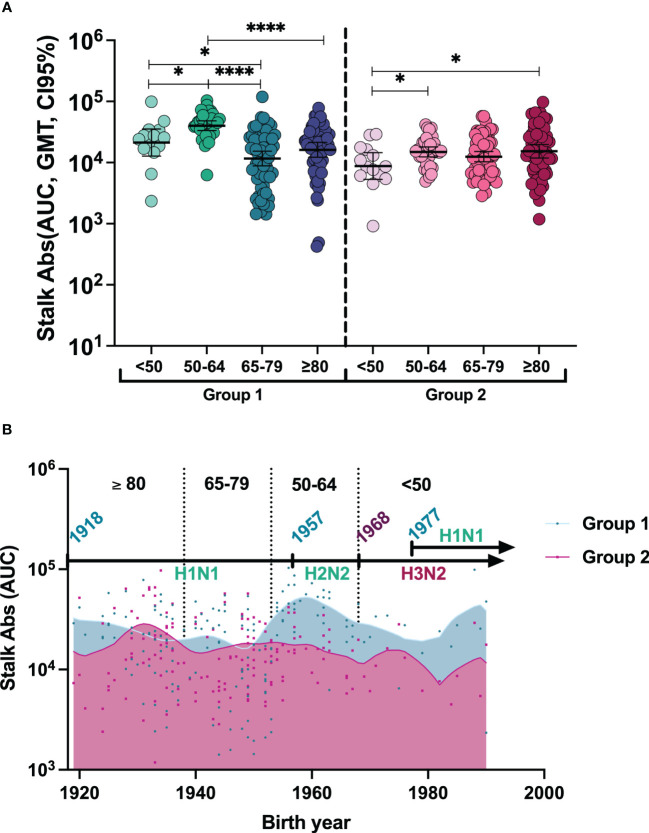
**(A)** Individual anti-stalk antibodies and geometric mean titer (GMT, 95% CI) before vaccination against HA groups 1 and 2 in all groups. To compute differences between age cohorts: The two-tailed p-values were calculated with the Brown–Forsythe and Welch ANOVA test adjusted by controlling the false discovery rate (FDR) with the two-stage linear procedure of Benjamini, Krieger, and Yekutieli for multiple comparisons. *P <0.05, ****P <0.0001. **(B)** Stalk antibody pre-immunity trend based on birth year. To represent anti-stalk antibodies based on theoretical first exposures to A viruses, individual antibody levels of patients based on their birth year were represented against both HA groups, and Lowess curves were designed with medium smoothing, taking 10 points in the smoothing window.

To better understand the baseline antibody landscape, we first profiled the pre-existing immunity before vaccination. For this, we investigated the levels of anti-stalk antibodies against HA groups 1 and 2 using reassortant viruses containing an exotic HA head domain and an exotic NA to whom humans should not have specific antibodies and a conserved stalk from human pandemic H1N1 virus A/California/04/09 and H3N2 virus A/HongKong/4801/2014 (groups 1 and 2, respectively). Purified viruses were then used to perform ELISA assays. To improve visualization, the levels of anti-stalk antibodies of each individual together with the geometric mean titer (GMT, CI95%) at day 0 are shown in **Figure 1A** and **Supplementary Table 1**. All vaccinees presented anti-stalk antibodies against both phylogenetic groups. The stalk antibody levels against HA group 1 in the 50–64-year-old group were significantly higher compared to <50-year-old, ≥80-year-old, and 65–79-year-old groups. Additionally, antibody levels in the <50-year-old group were also significantly higher than those in the 65–79-year-old group. In contrast, stalk antibodies against group 2 were lower in the <50-year-old cohort compared to the 50–64-year-old and the ≥80-year-old groups. Since different years of birth could influence previous exposure to different influenza viruses and therefore pre-existing immunity to influenza A viruses (IAVs), we next investigated the levels of immunity in the context of historical IAV circulation. In order to understand whether first exposure to influenza A viruses could have had an impact on preexisting immunity of anti-stalk HA group 1 versus HA group 2 antibodies, we analyzed antibody levels in the context of birth year. To do so, anti-stalk antibody levels based on birth year against each HA group were plotted, and Lowess curves were generated (**Figure 1B**). The timeline and emergence of different influenza A viruses and their circulation over the years are indicated to represent the likelihood of group 1 or 2 HA influenza primary infection. There is not a clear pattern, indicating that the likelihood of first exposure to either HA group virus could have an influence on pre-existing immunity. However, higher antibody levels against HA group 1 were found in younger adults when compared to older age groups, while an increasing tendency with age was found for group 2 anti-stalk antibodies (**Figure 1A**).

**Figure 2:**
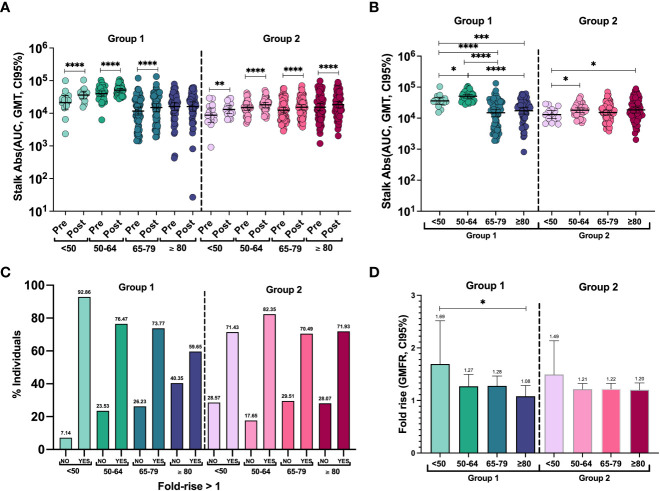
**(A)** Individual antibody levels and geometric mean titer (GMT, 95% CI) before and after vaccination against groups 1 and 2 of HAs in each group. The two-tailed p-values were calculated with the Wilcoxon matched pairs signed rank test. **P <0.01, ****P <0.0001. **(B)** Individual anti-stalk antibodies and geometric mean titer (GMT, 95% CI) after vaccination against HA groups 1 and 2 in all groups. To compute differences between cohorts, the two-tailed p-values were calculated with the Brown–Forsythe and Welch ANOVA test adjusted by controlling the false discovery rate (FDR) with the two-stage linear procedure of Benjamini, Krieger, and Yekutieli for multiple comparisons. *P <0.05, ***P <0.001, ****P <0.0001. **(C)** Percentage of responders and non-responders to seasonal influenza vaccination against groups 1 and 2 of HAs in each group. Responders are considered to have a fold rise of anti-stalk antibodies >1. **(D)** Geometric mean fold rise (GMFR, 95% CI) of stalk antibody levels. To compute differences between cohorts: The two-tailed p-values were calculated with the Brown–Forsythe and Welch ANOVA test adjusted by controlling the false discovery rate (FDR) with the two-stage linear procedure of Benjamini, Krieger, and Yekutieli for multiple comparisons. To compute differences in antibody levels within the same cohort: The two-tailed p-values were calculated with the Wilcoxon matched pairs signed rank test. *P <0.05.

To characterize the antibody response to both groups after influenza vaccination, we next investigated anti-stalk antibody levels at day 28. A modest but significant increase compared with baseline levels was observed in all age groups, except for anti-group 1 stalk antibodies in the ≥80-year-old group (**Figure 2A**). Post-vaccination stalk antibody titers against group 1 were significantly higher in the 50–64-year-old group compared to the other groups. Again, the titers in the <50-year-old group were significantly higher than those in the 65–79-year-old group and the ≥80-year-old group. Stalk antibodies against group 2 showed the same profile as before vaccination and were lower in the <50-year-old group compared to the 50–64-year-old and ≥80-year-old groups (**Figure 2B**) (**Supplementary Table 1**).

To quantify the induction of an antibody response to vaccination, we next calculated the geometric mean fold rise (GMFR). Despite only a few patients displaying a higher than 4-fold increase, more than 70% of the individuals in all age groups showed some level of increase in anti-stalk antibodies, except for the ≥80-year-old group, where 59.65% of them showed an increase in group 1 anti-stalk antibodies (**Figure 2C**). The distribution of fold-rise levels is also detailed in **Supplementary Figure 2**. Adjusted two-tailed p-values for multiple comparisons after Brown–Forsythe and Welch ANOVA were used to compare GMFR against each group (**Figure 2D**). Overall responses were similar for both HA groups 1 and 2. However, there was a significantly higher response to group 1 in the youngest patients compared to the oldest (p = 0.0382). No differences were found in fold induction levels in group 2 HAs between different age groups or when comparing the responses of groups 1 and 2 within age groups.

## 4 Discussion

The results of our study indicate that (a) pre-existing HA stalk immunity against phylogenetic group 1 is higher in younger populations; (b) seasonal influenza vaccines can moderately (on average less than two times) boost cross-reactive antibody responses against the stalk domain of both group 1 and group 2 HA viruses; and (c) age and previous exposures could impact responses to conserved epitopes, such as those against the stalk.

Responses to the influenza virus in adults are variable and complex as they are influenced by many factors (17). Humoral responses to the influenza virus rely on individual histories of exposure to the virus and are mainly targeted at the HA head (18). However, rapid evolution and antigenic drift make them of lesser importance when we talk about lifelong protection. In contrast, anti-stalk antibodies target more conserved epitopes and provide cross-reactive protection against different strains of the same phylogenetic group, resulting in an attractive approach to new vaccine development (19). Additionally, they have been recently associated as an independent correlate of protection in the case of group 1 HAs (20). Those antibodies are elicited most effectively after natural infection or vaccination with antigenically diverse strains. Our results showed higher baseline antibody levels against group 1 HAs in individuals <65 years old, in particular those 50–64 years old. This is in contrast with previous findings that suggest that they tend to increase with age (21–24). In the present study, only in group 2 HAs, anti-stalk antibodies seemed to increase with age. Although those studies included different age groups and vaccines, their results agree with ours in finding better responses in young adults <50 years old against group 1 HAs (21, 22) and no differences in responses in group 2 HAs (22).

On the other hand, unlike group 1 Has, of which several different antigenic strains have circulated in humans (H1N1, H2N2, and H1N1pdm09), antigenically similar group 2 HA viruses have circulated in humans since 1968 (25) (**Supplementary Figure 3**). It has been shown that divergent strains are more likely to drive the expansion of cross-reactive antibodies against more conserved epitopes, such as the HA stalk, than similar ones (24). It is possible that the lack of stimulus from substantially divergent strains is responsible for the lower magnitude of antibodies against group 2 HAs in younger individuals. These results align with a previous longitudinal study where the highest levels of group 1 HAs were found in individuals exposed to the most diverse group 1 viruses (26). Additionally, responses against the head domain have also been described as being lower against phylogenetic group 2 (27). Therefore, the lower magnitude in these age groups could also suggest that group 2 HA viruses are less immunogenic.

The term antigenic seniority or antigen imprinting describes how influenza antibody responses in humans are shaped by the first encounters in life, usually at an early stage, and upon repeated exposure, either by infection or vaccination. This concept is commonly known as the original antigenic sin. Humoral responses after natural infection induce broader and longer-lasting responses than after vaccination (28). However, responses to vaccination are not equal and depend on the immunodominance of different epitopes as well as the age of individuals (12). In fact, it has been shown that antibody responses against the stalk domain are suppressed in favor of the head domain with currently licensed influenza vaccines (29, 30). Not many studies attribute an increase in stalk antibodies to seasonal vaccination (31). Although our previous results confirmed that most responses are directed against the HA head (12), here we show that a modest but significant rise in stalk titers can be found in most individuals after influenza vaccination. Also, these responses were higher in younger populations despite receiving a non-adjuvanted influenza vaccine, in contrast to the adjuvanted vaccine received by older individuals. This reduction in immune responses, known as immunosenescence, impairs antibody avidity and B- and T-cell responses to vaccination as we age (32, 33). This phenomenon could be one of the reasons for the reduction in baseline levels with age in the case of group 1 HA anti-stalk antibodies. However, we cannot explain why group 2 HA responses seem not to be affected by immunosenescence in a similar way. Nevertheless, responses in the younger populations are more uniform, while responses in the elderly seem to have higher variability. This could be explained by the variability in the degree of immunosenescence, which has been recently proposed not to be a strict decline but a dynamic balance that might be necessary for an adequate response to known antigens but detrimental to responses to new antigens in most circumstances (34).

Our analysis by birth year did not show a pattern according to the likely first exposure to each HA group of viruses in our age groups. However, the group of 50–65 year olds who could have first encountered A(H2N2) had higher pre-existing immunity, while the elderly (≥65 years old) showed unexpected results with lower baseline anti-stalk antibody levels against this group and like those against HA group 2 levels. These findings could be explained by immunosenescence in the elderly population. However, further studies should be performed to understand the effect of imprinting on age.

To conclude, our results show that, in general, modest responses are elicited against both HA groups 1 and 2 and that consecutive exposures to substantially different strains drive responses against the HA stalk domain. This concept is already being used for universal vaccine approaches that aim at eliciting broad, long-lasting, cross-reactive protection with chimeric HA designs (35). However, our findings suggest that immunosenescence, especially in older patients, could drive lower responses to seasonal vaccination. Therefore, strategies that aim to enhance immune responses in the elderly should be considered for future vaccine designs (36).

Our study has several limitations. First, it was designed as a sero-epidemiological study of vaccine responses, and only serum samples were available. Second, the cohorts analyzed differed in the type of vaccine recommended by the Spanish health agencies and sometimes were not strictly followed. Third, the lack of information on previous exposures to influenza virus makes it difficult to interpret results, although the likelihood of priming could be inferred from the year of birth.

## Data availability statement

The raw data supporting the conclusions of this article will be made available by the authors, without undue reservation.

## Ethics statement

This research was performed according to the Declaration of Helsinki and was approved by the Ethics Committee of East-Valladolid health area under the code PI 21-2314. The patients/participants provided their written informed consent to participate in this study.

## Author contributions

TA and AG-S conceived, designed, and supervised the study. TA provided training to LS-dP. WS and PP generated the viruses. LS-dP performed the experiments, including the growth of viral stocks and ELISAS. Samples were provided by IS-M, JE, and RO. LS-dP and TA analyzed data, wrote the manuscript, and prepared the figures. TA, AG-S, and PP provided reagents, methods, and expertise. TA, AG-S, JE, and IS-M supervised the study. All authors contributed to the article and approved the submitted version.
