# Supplementary material for: Group 1 and group 2 hemagglutinin stalk antibody response according to age
Source: Front Immunol. 2023 May 29;14:1194073. doi: 10.3389/fimmu.2023.1194073 (PMC10258341; doi:10.3389/fimmu.2023.1194073)
Supplement: Supplementary file 1 [file DataSheet_1.docx]

Supplementary Material

**GROUP 1 AND GROUP 2 HEMAGGLUTININ STALK ANTIBODY RESPONSE ACCORDING TO AGE**

**Laura Sánchez-de Prada, Iván Sanz-Muñoz, Weina Sun, Peter Palese, Raúl Ortiz de Lejarazu, José María Eiros, Adolfo García-Sastre* and Teresa Aydillo***

*** Correspondence:** Teresa Aydillo Adolfo García-Sastre
 [teresa.aydillo-gomez@mssm.edu](mailto:teresa.aydillo-gomez@mssm.edu) [adolfo.garcia-sastre@mssm.edu](mailto:adolfo.garcia-sastre@mssm.edu)

# Supplementary Methods

**1.1 Generation of the cH14/3N5 virus**

The cH14/3N5 virus had an HA stalk derived from an H3N2 virus A/Hong Kong/4801/2014 combined with an exotic H14 head domain A/mallard/Gurjev/263/1982 and an exotic N5 from A/mallard/Sweden/86/2003 (supplementary methods) To rescue the cH14/3, HA and NA sequences were obtained by gBlock DNA synthesis via IDT and cloned into pDZ plasmids by in-Fusion cloning (Takara) to obtain pDZ-cH14/3 and pDZ-N5 rescue plasmids. HEK 293T cells were transfected with pDZ-cH14/3, pDZ-N5 and pRS-6Seg PR8 [1] for 48 hours. Cells and supernatants were harvested and briefly homogenized by several syringe strokes. Two hundred microliters of the homogenized cell and supernatant mixture were injected into each 8-day old embryonated chicken egg, which was incubated at 37ºC for 2 days. Eggs were then cooled at 4ºC overnight. Allantoid fluids were harvested, and the rescue of virus was determined by HA assay. Virus was plaque-purified on MDCK cells and further amplified in 10-day old embryonated chicken eggs. Viral RNA extraction was performed using QIAamp Viral RNA mini kit. The HA and NA DNA segments were amplified using SuperScriptTM III one-Step RT-PCR System with PlatinumTM Taq DNA polymerase. The sequences of the HA and NA segments were confirmed by Sanger sequencing (Genewiz).

**1.2. Virus purification in sucrose gradient**

To purify the viruses cultured in allantoid fluid an ultracentrifugation in sucrose gradient was performed. To do so, 5mL of 30% sucrose was added at the bottom of an ultracentrifuge tube and the virus is added slowly on top of it and centrifuge at 25,000 rpm and 4ºC for 2 hours. Then aspirate the supernatant and resuspend the pellet in PBS and centrifuge again at 25,000 rpm and 4ºC for 1,5 hours. Aspirate the supernatant and resuspend in the desired volume. Then, the amount of protein extracted was measured with a Fluorometer (Qubit 4, Thermofisher Scientific).

**1.3. Enzyme-linked Immunosorbent Assay (ELISA)**

To measure anti-stalk antibodies in human serum flat-bottom 96-well plates (Immulon 4 HBX; Thermo Fisher Scientific) were coated with 100 µL/well of the virus in phosphate-buffered saline solution (PBS; pH 7.4; Gibco, NY, USA) at a concentration of 5 mcg/mL each for cH6/1 and cH14/3 and incubated at 4ºC overnight. Next, plates were washed 3 times with washing buffer (PBS containing 0.1% Tween-20; Fisher Scientific). Plates were incubated 1.5 hours at room temperature with 220 µL/well blocking solution (washing buffer containing 0.5% non-fat powdered milk, Boston BioProducts, and 3% goat serum, GIBCO). Blocking solution was removed and 100 µL of two-fold-diluted serum samples starting from 1:800 initial dilution was added to each well and incubated for 1.5 hours at room temperature. Plates were then washed 4 times with washing buffer and 50 µL of a peroxidase-conjugated antihuman IgG (Fc-specific) monoclonal antibody (Sigma) was added at a final concentration of 1:20,000 in blocking solution. After washing, 100 µL of peroxidase substrate (3,30,5,50-Tetramethylbenzidine, TMB, Rockland) was added and incubated in the dark at room temperature for 30 min. The reaction was stopped with 4 N H2SO4 solution (ThermoFisher Scientific). The absorbance was measured at 450 nm with a plate spectrophotometer (Synergy H1 hybrid multimode microplate reader, Biotek). Optical density (OD) for each well was calculated by subtracting the average background plus three standard deviations. Area under the curve (AUC) was computed using GraphPad Prism v.10 software.

# Supplementary Figures and Tables

## Supplementary Figures


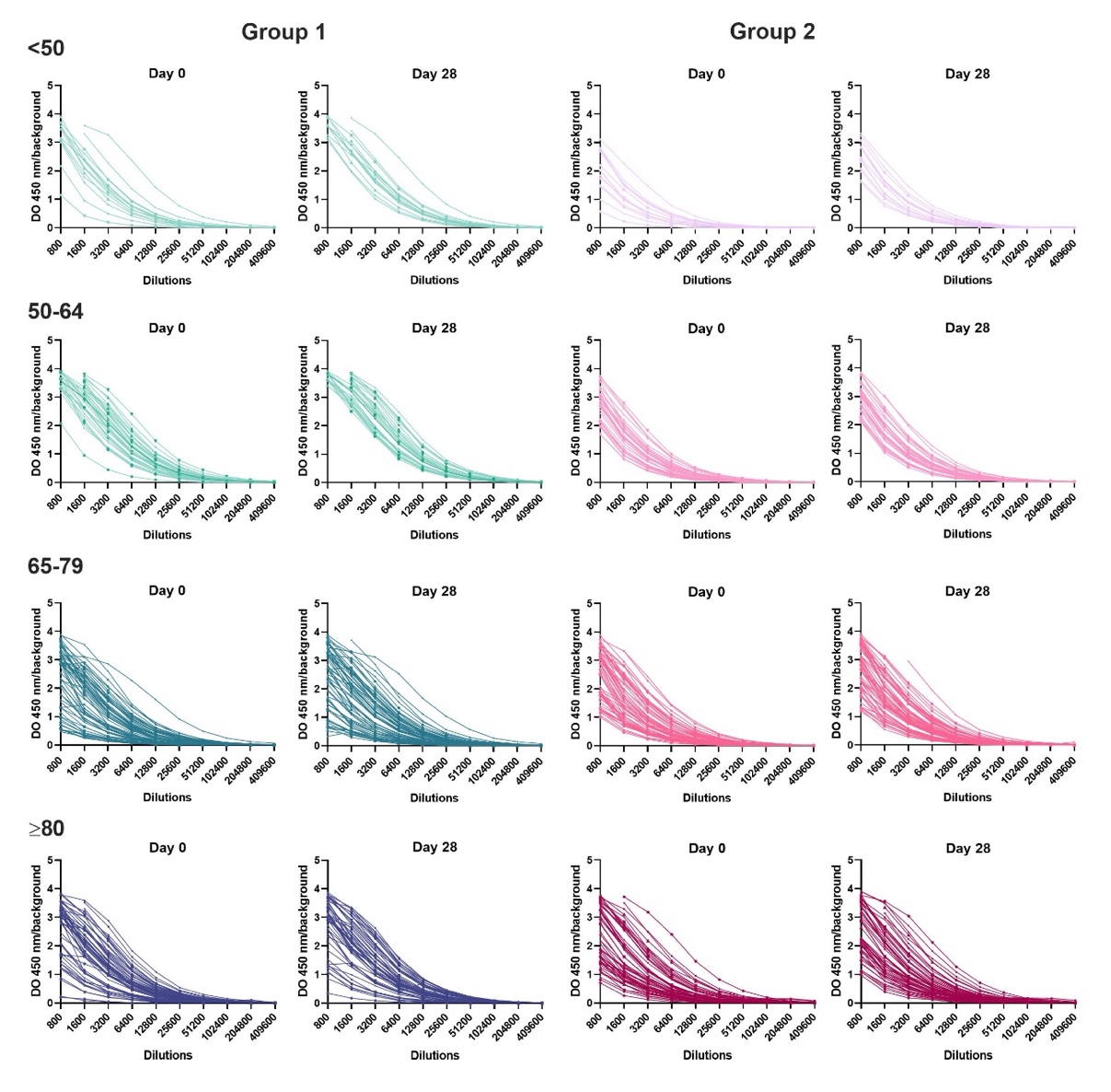


**Supplementary Figure 1.** Binding profile of the serial dilution curve for each individual serum sample collected at day 0 and 28 in each cohort ELISAs assays were performed for group 1 and group 2 of HAs. Area-under-the-curve (AUC) values were computed by plotting normalized optical density (OD) values against the reciprocal serum sample dilutions and used to quantify antibody titers for each individual serum.


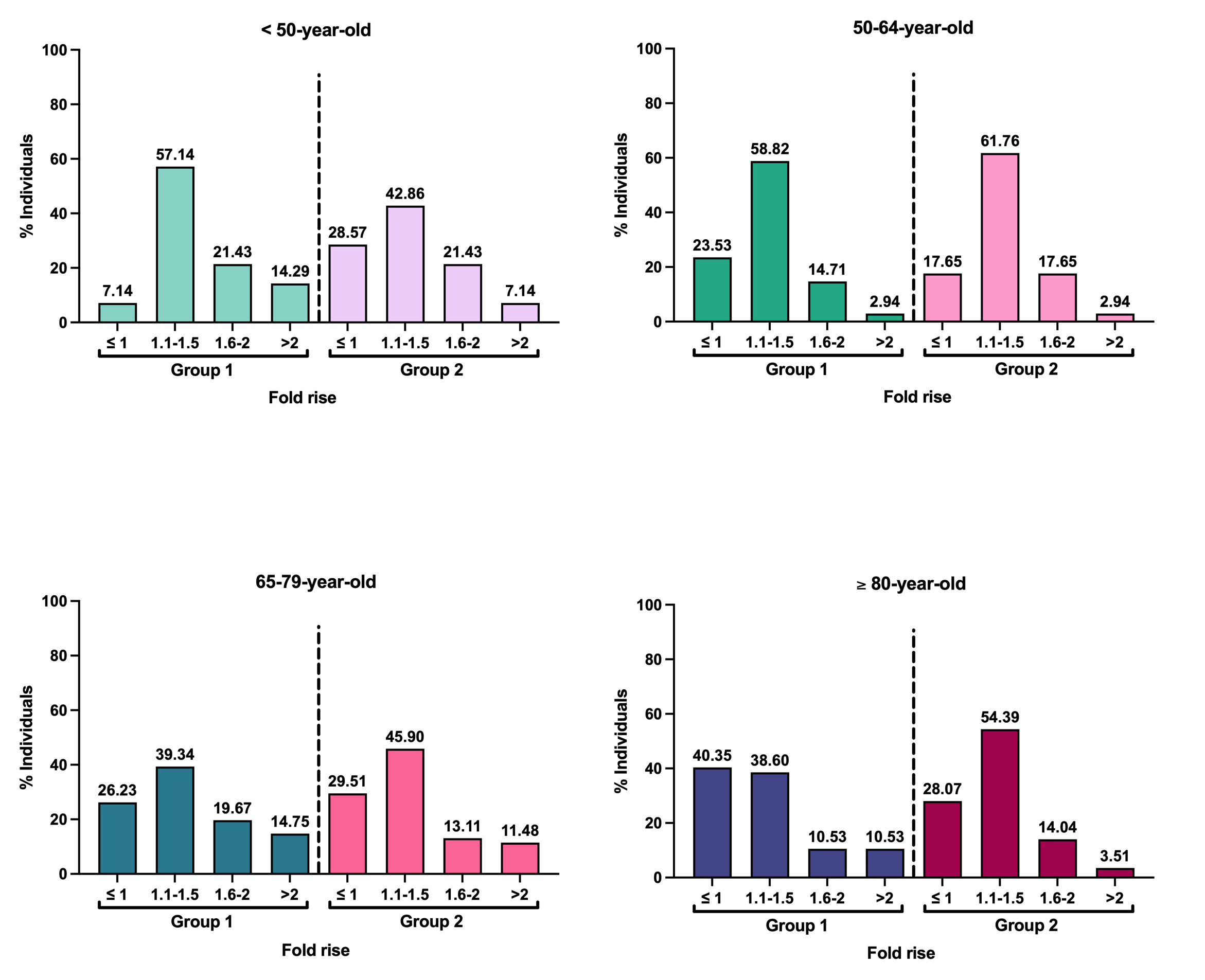


**Supplementary Figure 2.** Distribution of the fold-rise levels achieved by individuals against group 1 and group 2 of HAs after seasonal influenza vaccination in each age group.


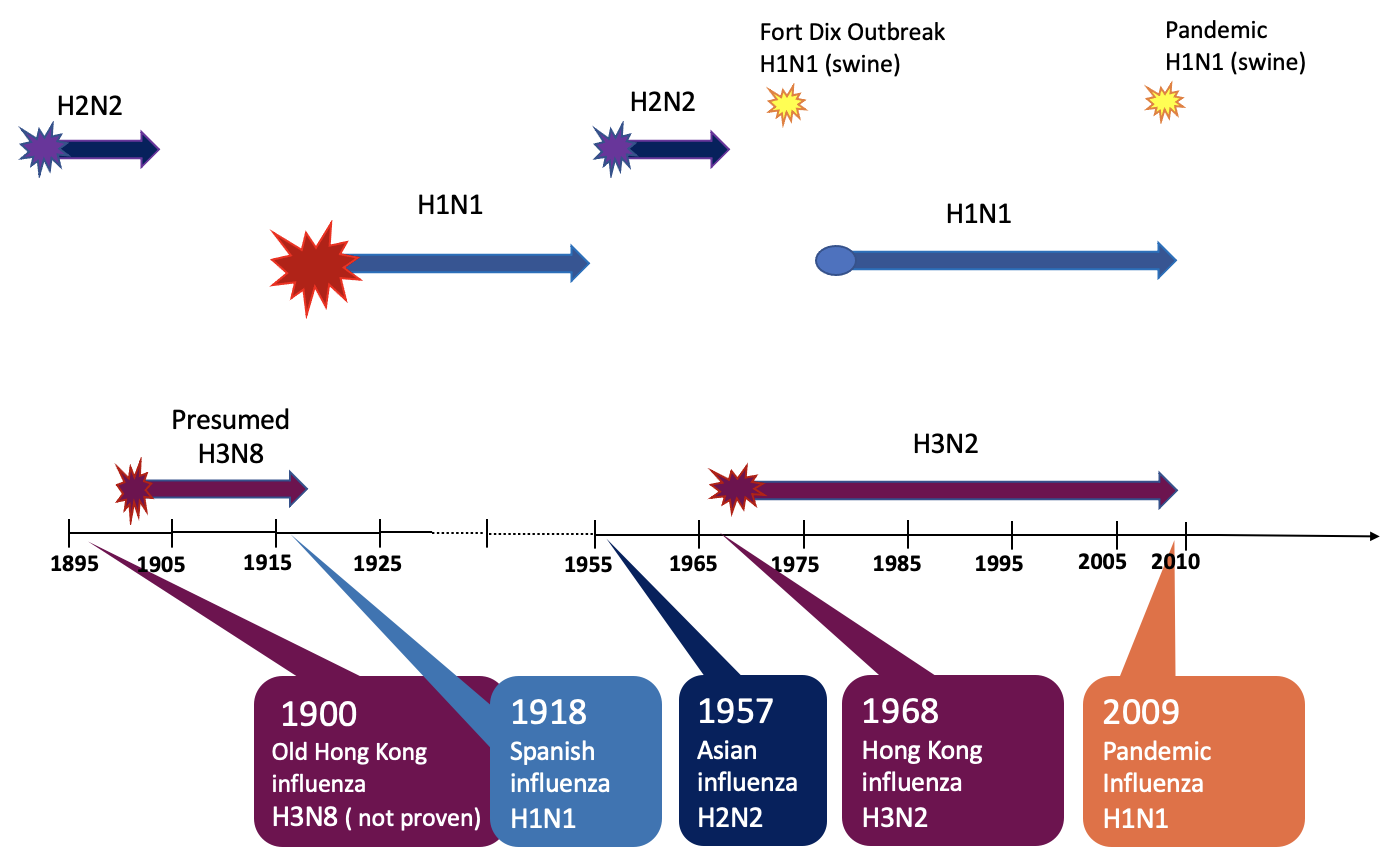


**Supplementary figure 3.** History of influenza pandemics and circulation of XX and XXI centuries. Adapted from: https://www.eurosurveillance.org/content/10.2807/ese.15.01.19458-en#html_fulltext

## Supplementary Tables

**Supplementary table 1**. Antibody levels and fold antibodies rise against group 1 and group 2 of HAs before and after vaccination against influenza.

|  | **Cohorts** | | | | | | | |
| --- | --- | --- | --- | --- | --- | --- | --- | --- |
|  | **<50** | | **50-64** | | **65-79** | | **≥80** | |
|  | **Group 1** | **Group 2** | **Group 1** | **Group 2** | **Group 1** | **Group 2** | **Group 1** | **Group 2** |
| **Day 0 GMT** | 21,255 | 8,765 | 40,138 | 14,909 | 11,647 | 12,491 | 16,137 | 15,319 |
| **(CI95%)** | (12,766-35,389) | (5,286-14,534) | (33,415-48,214) | (12,345-18,005) | (8,815-15,388) | (10,280-15,176) | (12,204-21,337) | (11,865-19,778) |
| **Day 28 GMT** | 36,162 | 13,091 | 50,897 | 18,113 | 14,975 | 15,244 | 17,385 | 18,42 |
| **(CI95%)** | (27,581-47,411) | (9,708-17,654) | (44,576-58,115) | (15,239-21,529) | (11,473-19,544) | (12,776-18,187) | (13,557-22,294) | (14,698-23,086) |
| **GMFR** | 1.69 | 1.49 | 1.27 | 1.21 | 1.28 | 1.22 | 1.08 | 1.20 |
| **(CI95%)** | (1.14-2.52) | (1.04-2.14) | (1.08-1.50) | (1.12-1.32) | (1.11-1.46) | (1.12-1.33) | (0.91-1.28) | (1.08-1.33) |

GMT: geometric mean titre; CI 95%: confidence interval 95%; GMFR: geometric mean fold rise

## References

[1] Fulton BO, Sun W, Heaton NS, Palese P. The Influenza B Virus Hemagglutinin Head Domain Is Less Tolerant to Transposon Mutagenesis than That of the Influenza A Virus. J Virol 2018;92. https://doi.org/10.1128/JVI.00754-18.
